# Catalase-Based Modified Graphite Electrode for Hydrogen Peroxide Detection in Different Beverages

**DOI:** 10.1155/2016/8174913

**Published:** 2016-12-18

**Authors:** Giovanni Fusco, Paolo Bollella, Franco Mazzei, Gabriele Favero, Riccarda Antiochia, Cristina Tortolini

**Affiliations:** ^1^Department of Chemistry and Drug Technologies, Sapienza University of Rome, Rome, Italy; ^2^Department of Chemistry, Sapienza University of Rome, Rome, Italy

## Abstract

A catalase-based (NAF/MWCNTs) nanocomposite film modified glassy carbon electrode for hydrogen peroxide (H_2_O_2_) detection was developed. The developed biosensor was characterized in terms of its bioelectrochemical properties. Cyclic voltammetry (CV) technique was employed to study the redox features of the enzyme in the absence and in the presence of nanomaterials dispersed in Nafion® polymeric solution. The electron transfer coefficient, *α*, and the electron transfer rate constant, *k*_*s*_, were found to be 0.42 and 1.71 s^−1^, at pH 7.0, respectively. Subsequently, the same modification steps were applied to mesoporous graphite screen-printed electrodes. Also, these electrodes were characterized in terms of their main electrochemical and kinetic parameters. The biosensor performances improved considerably after modification with nanomaterials. Moreover, the association of Nafion with carbon nanotubes retained the biological activity of the redox protein. The enzyme electrode response was linear in the range 2.5–1150 *μ*mol L^−1^, with LOD of 0.83 *μ*mol L^−1^. From the experimental data, we can assess the possibility of using the modified biosensor as a useful tool for H_2_O_2_ determination in packaged beverages.

## 1. Introduction

In recent years, many researchers focused their activity on developing new tools to detect H_2_O_2_, not only as an oxidases reaction byproduct but also as a conservative compound in food and drugs [1, 2]. Indeed, hydrogen peroxide finds significant employment in industrial processes as an oxidant [3]: in particular, hydrogen peroxide is released into the environment in either small or large amounts, since it is used as oxidant, whitening, or sterilant tool in packaging materials owing to its sporicidal and bactericidal features [4–6]. Nevertheless, high H_2_O_2_ concentrations would be dangerous for human beings [7–10].

Several analytic methods as chemiluminescence [11–17], photometry [18], fluorimetry [19–21], titrimetry [22, 23], spectrophotometry [23–26], high-performance liquid chromatography (HPLC) [27], and especially electrochemistry [3, 28–37] are reported in the literature for detection of hydrogen peroxide.

The electrochemical techniques provide some interesting advantages in comparison to the other ones mentioned above like fast, specific, and cheap monitoring of hydrogen peroxide [37–43]. The direct reduction of H_2_O_2 _at a bare sensor is not suitable for analytical measures due to its slow kinetics and high potentials required for redox reactions [44]. To overcome these problems, several modified electrochemical sensors were developed. Electrochemical biosensors based on the biocatalytic activity of immobilized enzymes towards the substrate H_2_O_2 _are helpful because of their high sensitivity, selectivity, and ease of use [45, 46]. Some authors, in the recent years, have applied different modified biosensors, based on various redox proteins, to realize interesting tools for the monitoring of H_2_O_2_ [45, 47–55].

Catalase (CAT) belongs to oxidoreductase family class and has a heme prosthetic group at its active site with ferric ion (Fe(III)) [48, 50, 56–59]. The catalytic ability of CAT to reduce hydrogen peroxide was used in the developing of biosensors [50, 56, 60]. To investigate CAT catalytic activity, it is important to study its capacity to perform direct electron transfer (DET) to the electrode surface. It is usually difficult to observe the DET because the heme groups are buried deeply inside in the large structure of the protein [61, 62]. Also, denaturation of the redox protein could occur on the sensor surface due to the immobilization method and to the matrix composition. To overcome these problems and promote the DET carbon nanotubes (CNTs), modified electrodes are widely employed as support for the physical immobilization of biological molecules to promote the DET thanks to their high surface/volume ratio and conductivity and also to enhance sensors and biosensors performances [63–67]. A drawback on the use of CNTs to modify electrode surface is their insolubility [68, 69]. However, some authors have obtained good results in the CNTs modification of the electrode surface by using polymers as dispersing support [70, 71]. Nafion is a perfluorinated polymer resistant to chemical attack and the CNTs dispersion in its film has been investigated [72–74].

In the present study, we report the development of a biosensor for H_2_O_2 _monitoring based on the immobilization of catalase in a Nafion film containing dispersed functionalized MWCNTs-COOH. The Nafion film ensures efficient immobilization of the protein in its native configuration. The DET of catalase was investigated either on modified or on bare electrode to identify the optimal conditions for H_2_O_2_ detection. In view of the possible practical application, the same modification steps were performed on screen-printed electrodes (SPEs) with a working electrode based on mesoporous graphite (MG-SPE). Finally, the obtained biosensor was applied for the determination of hydrogen peroxide in beverages samples.

## 2. Experimental

### 2.1. Materials and Reagents

Catalase from bovine liver (CAT, EC 1.11.1.6, activity ≥ 10,000 U mg^−1^ protein) was supplied by Sigma-Aldrich (Switzerland) and stored at −20°C. All chemicals used were of analytical grade. In particular, Na_2_HPO_4_, NaH_2_PO_4_, HOC(COOH)(CH_2_COOH)_2_, KCl, (K_3_[Fe(CN)_6_]), Nafion 117 solution (NAF, purum, ~5% solution in a mixture of lower aliphatic alcohols and water), CH_3_CH_2_OH (~96% v/v), and H_2_O_2_ (30 wt.% in H_2_O) were purchased from Sigma-Aldrich (Switzerland). High purity deionized water (resistance: 18.2 MΩ  × cm at 25°C; TOC: <10 *μ*g L^−1^) obtained from a Millipore Direct-Q 3 UV system (France) was used throughout the experiments. The working solutions were prepared by diluting the stock solution with 0.1 mol L^−1^ phosphate buffer solution and 0.1 mol L^−1^ KCl, pH 7.0 (PBS buffer solution), and then deoxygenated by bubbling N_2 _for about 20 min. Multiwalled carbon nanotubes modified with carboxylic groups (MWCNTs-COOH) were obtained from DropSens (Spain).

### 2.2. Electrochemical Measurements

All electrochemical measurements were performed with *μ*-Autolab type III potentiostat (EcoChemie, Netherlands) controlled using the GPES Manager program (EcoChemie, Netherlands) at room temperature in N_2_ atmosphere. Batch electrochemical experiments were performed in a 5 mL thermostated glass cell (model 6.1415.150, Metrohm, Switzerland) containing PBS buffer solution, with a conventional three-electrode system. Different working electrodes were used, in particular glassy carbon electrode (GCE, cat. 6.1204.300, Metrohm, Switzerland, *ϕ* = 3 mm) and a mesoporous graphite screen-printed electrode (MG-SPE, model DRP-110MC, *ϕ* = 4 mm, DropSens, Spain). A saturated calomel electrode (SCE, cat. 303/SCG/12, Amel Instruments, Italy) as the reference electrode and a carbon rod (cat. 6.1248.040, Metrohm, Switzerland) as the counter one were employed. For SPEs, the counter electrode was carbon and the reference one was silver, respectively. All the reported potentials are referred to as saturated calomel electrode (*E* = 0.241 V versus NHE). All pH measures were performed using a digital pH meter (827 pH lab, Metrohm, Italy). The morphology of the samples was observed using high-resolution field emission scanning electron microscopy (HR FESEM, Zeiss Auriga Microscopy) equipped with Microanalysis EDS ≤ 123 Mn-K*α* eV (Bruker).

### 2.3. Procedures

The GCE surface was polished with 0.3 and 0.05 *μ*m alumina slurry on polishing silk cloth (SIEM, Italy) and rinsed with deionized water. Then, the electrode was sonicated in deionized water to remove trace of alumina from the surface (Sonicator AU-32, ArgoLab, Italy).

The physical immobilization of the enzyme was realized by dropping onto the working electrode surface 2 *μ*L of 0.5 wt.% Nafion solution containing 1 mg mL^−1^ of redox protein either in the presence or in the absence of 1 mg mL^−1^ of MWCNTs-COOH. The electrode surface was finally air-dried for about 20 min at room temperature. The biosensors were stored in PBS buffer solution at 4°C before use.

The analysis protocol of real beverages is described as follows: 2.5 mL of different beverages sample was diluted to 10 mL with PBS buffer solution. Then, a certain amount of H_2_O_2_ (15 *μ*mol L^−1^) was added and the solutions were deoxygenated. Then, the samples were analyzed directly by cyclic voltammetry (CV) method and finally the recoveries were evaluated. For the study of pH dependance, the McIlvaine buffer was used at different pH values.

## 3. Results and Discussion

### 3.1. Electrochemical Characterization of Glassy Carbon Electrode after Steps of Modification

The effect on the improvement of electrochemical performances by using nanomaterials as MWCNTs-COOH was evaluated with cyclic voltammetry measurements of the electroactive area (*A*_*e*_) and of the heterogeneous standard rate constant (*k* ^0^) of the different electrodes. The cyclic voltammograms (not shown) were recorded in a solution of 1. 1 mmol L^−1^ potassium ferricyanide in PBS buffer solution. *A*_*e*_ was determined from the Randles-Sevčik equation: *I*_*p*_ = 2.686 × 10^5^ *n* ^3/2^ *A*_*e*_ *D* ^1/2^C*v* ^1/2^ [95], where *I*_*p*_ is current in amps (A), *n* is number of electrons transferred of K_3_[Fe(CN)_6_] by cyclic voltammetry (CV) in the redox event (usually 1), *A*_*e*_ is electroactive area (cm^2^), *D* is diffusion coefficient (7.6 × 10^−6^ cm^2^ s^−1^), *C* is concentration (mol cm^−1^), and *v* is scan rate (Vs^−1^). *k* ^0^ was calculated by an extended method [96], a combination of Nicholson [97] and Klingler and Kochi treatments [98], by CV data using the same solution described above, in the scan rate range 5–100 mV s^−1^.

By comparing the results (see Table 1) arising from the several modification steps of the sensor, two aspects can be pointed out: (i) the parameters obtained for the Nafion modified sensor (NAF-GCE) are lower than both the bare sensor (bare-GCE) and the nanomaterial modified sensor (NAF-MWCNTs-COOH-GCE): presumably, this is due to the Nafion film that hinders the charge transfer and slows down the substrate rate towards the sensor surface; (ii) the use of carbon nanotubes enhances hugely the electrochemical signal increasing *A*_*e*_ (about 4 and 8 times compared to the bare-GCE and NAF-GCE, resp.) and improves *k* ^0^ of the ferricyanide ion towards the sensor surface despite the ion exchange polymer presence (about 1.5 and 2 times compared to the bare-GCE and NAF-GCE, resp.): this could be ascribed to their excellent properties of increasing area/volume ratio and high electron conductivity and of facilitating the electron transfer [99–104]. The association of these nanomaterials with Nafion (as solubilizing agent) does not impair the electrocatalytic features of carbon nanotubes. This aspect was also observed in our previous work where the use of NAF/MWCNTs composite film has greatly increased the transfer charge rate [105].

### 3.2. Biosensor Voltammetric Behavior before and after Nanomaterial Modification

**Figure 1:**
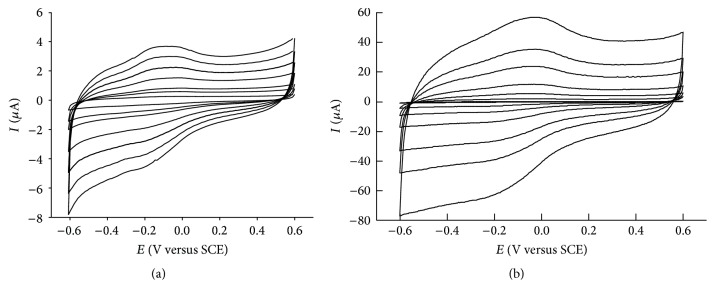
CVs for NAF-GCE-CAT (a) and NAF-MWCNTs-COOH-GCE-CAT (b) at different scan rates (10–500 mV s^−1^) in deoxygenated PBS buffer solution.

The comparison of electrocatalytic performances was evaluated by using catalase as model redox protein and comparing the voltammetric behavior (Figures 1(a) and 1(b)) measuring several electrochemical parameters (see Section 3.4). The catalase was immobilized by a Nafion film onto the GCE surface, in the absence and in the presence of MWCNTs-COOH; the electrochemical behavior of the modified electrodes has been investigated in N_2_ saturated PBS buffer solution, using CV. The cyclic voltammograms were recorded at NAF-GCE-CAT and NAF-MWCNTs-COOH-GCE-CAT modified GCEs in the potential range from 0.6 V to −0.6 V. In the absence of MWCNTs-COOH, catalase immobilized in a Nafion film onto GCE surface showed a quasi-reversible signal (see Figure 1(a)) with a midpoint potential of *E* ^0′^ = −128 mV; the separation of cathodic and anodic peak potential Δ*E*_*p*_ = 80 mV (at scan rates lower than 100 mV s^−1^) indicated a fast electron transfer reaction according to the literature [106]. For the other modified electrode, when the redox protein is in the presence of carbon nanotubes, CV experiments yielded evidence of a prominent increase (about 20 times) of faradic current (Figure 1(b)) and also an enhancement of electron transfer kinetic was observed at a constant amount of immobilized protein. In particular, *E* ^0′^ shifted to a more negative potential value (−140 mV) and Δ*E*_*p*_ was 70 mV, assuming that carbon nanotubes play an important role in the rising of the system reversibility.

### 3.3. Study of pH Dependence on the Modified Electrode

**Figure 2:**
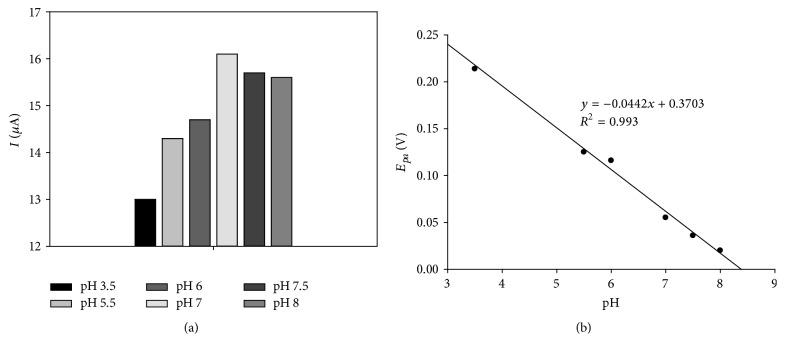
The effect of pH on the redox peak currents of NAF-MWCNTs-COOH-GCE-CAT in various buffer solutions with pH values 3.5, 5.5, 6.0, 7.0, 7.5, and 8.0 (a); *E*_*pa*_ versus pH plot (b).

The effect of pH solution on the modified NAF-MWCNTs-COOH-GCE-CAT electrode was also tested. In Figure 2(a), the peak currents at different pH values are shown. The maximum of anodic current occurred at pH 7.0. This value was consistent with that reported for catalase enzyme [60, 76–78]. Based on these results, pH 7.0 for PBS buffer solution was used as the optimal pH for further experiments. Also, the influence of pH solution on the oxidation peak potentials was investigated. The oxidation peak potential was reported versus solution pH values in the range 3.5–8.0 (Figure 2(b)). The obtained slope (0.044 V) suggests that the reaction at the electrode surface is accompanied by proton transfer. The slope value is slightly smaller than Nernst's value of 0.059 V pH^−1^ for the reaction of one electron coupled to one proton [76]. This is probably ascribable to the influence of protonation states of trans ligands of the heme iron and amino acids around the heme, or the protonation of H_2_O molecule coordinated to the coordinated iron [107, 108].

### 3.4. Cyclic Voltammetric Studies of Direct Electron Transfer of Catalase before and after Nanomaterial Modification of the Biosensor

**Figure 3:**
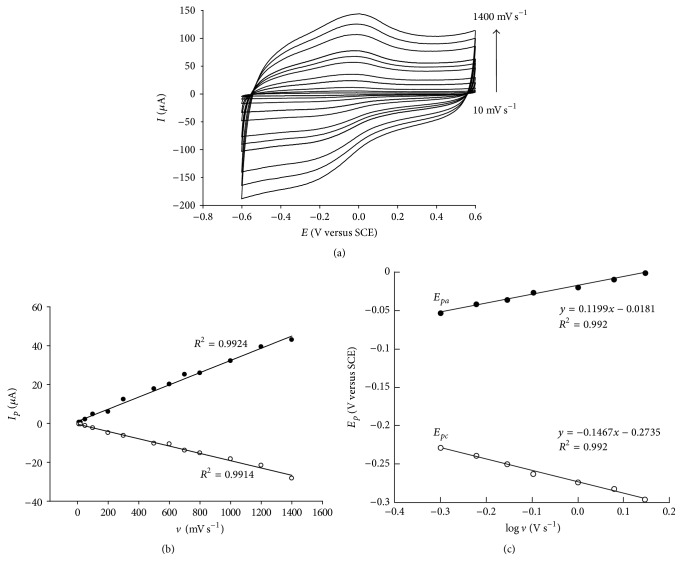
CVs for NAF-MWCNTs-COOH-GCE-CAT in deoxygenated PBS buffer solution at various scan rates (a). Relationship between the anodic and cathodic peak currents and scan rates (b). Relationship between peak potential separation and logarithm of scan rates (c).

Figure 3(a) shows typical cyclic voltammograms of NAF-MWCNTs-COOH-GCE-CAT biosensor at different scan rates (10–1400 mV s^−1^). The dependence of peak currents and peak potentials on the scan rate is also observed in Figures 3(b) and 3(c), respectively. As is obvious from Figure 3(b), the peak currents change linearly with scan rate over a range of 10 to 1400 mV s^−1^ (with correlation coefficients of 0.9924 and 0.9914), as expected for thin layer electrochemistry [35, 109] and according to a surface-controlled process. The slope of corresponding log *I*_*p*_ versus log *v* linear plot, with a correlation coefficient of 0.9949, was found to be 1.115, very close to the theoretical slope 1 for thin layer voltammetry [109].

The surface concentration of electroactive redox protein (Γ) can be estimated using Faraday law (see (1)) and calculated from the slope of peak current/scan rate plot [76, 109]:

(1) $$\begin{array}{c} \Gamma =\frac{4I_{p}RT}{n^{2}F^{2}Av}, \end{array}$$

where *v* is the scan rate, *A* is the electrode surface area (0.07 cm^2^), *T* is the temperature, *n* is the number of electrons, and *R* and *F* are gas and Faraday constants, respectively. Thus, the average surface concentration Γ of catalase was found to be 4.76 × 10^−10^ mol cm^−2^, which indicates that the immobilized enzyme is in the form of an approximate monolayer on the surface of the modified electrode [63, 75].

Moreover, the peak-to-peak separation at a scan rate of 10 mV s^−1^ was approximatively 70 mV, indicating a quasi-reversible electron transfer process. Based on the Laviron theory [109], the transfer coefficient (*α*) and the electron transfer rate constant (*k*_*s*_) for immobilized catalase either in the absence or in the presence of nanomaterials can be estimated by measuring the variation of peak potential separation with scan rate (at higher scan rates, as shown in Figure 3(c)) and reported in Table 2.

Besides, by comparing our proposed biosensor to other similar ones in the literature [60, 77–83], all based on CAT modified GCEs by using MWCNTs, it is evident that the amount of our electroactive catalase is higher, probably due to the simple NAF/MWCNTs matrix that could increase the exposure extent of the heme group in the catalase enzyme (see Table 3). The formal potential *E* ^0′^ of our biosensor is much less negative than those proposed by other authors [63, 76–83, 108, 110]. The formal potential value is dependent on the protein structure [111, 112], so a change of the heme protein in the NAF/MWCNTs composite film results in a shift of *E* ^0′^ to positive potential values. Moreover, partial denaturation of the enzyme could cause heme leakage and then a negative shift of the redox peaks (change in the coordination sphere) [113].

### 3.5. Catalytic Activity of Catalase

**Figure 4:**
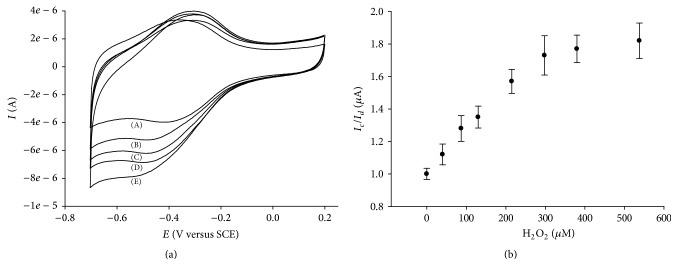
CVs of NAF-MWCNTs-COOH-GCE-CAT modified biosensor in the absence (A) and in the presence of 130 *µ*M (B), 215 *µ*M (C), 298 *µ*M (D), and 538 *µ*M (E) of the substrate H_2_O_2_ (a). Catalytic efficiency changes versus hydrogen peroxide, where *I*_*c*_ and *I*_*d*_ are the cathodic peak currents in the presence and in the absence of H_2_O_2_, respectively (b). Experimental conditions: deoxygenated PBS buffer solution, *v* = 50 mV s^−1^.

The voltammetric characterization of the hydrogen peroxide reduction by means of the developed NAF-MWCNTs-COOH-GCE-CAT biosensor was performed in PBS buffer solution, at a scan rate of 50 mV s^−1^ (Figure 4(a)).

An increase in the cathodic peak with the hydrogen peroxide concentration and a decrease in the anodic peak during the scan reversal have been observed. Conversely, in the absence of catalase, no current change has been detected by the NAF-MWCNTs-COOH-GCE electrode. From our experiments, we confirm the EC mechanism previously reported in the literature [77, 95]:

(2) Cat-FeIII+e−+H+⇌Cat-FeIIH+at  the  electrode  surfaceH2O2+Cat-FeIIH+⟶Cat-FeIII+H++H2Oin  solution

Figure 4(b) reports the catalytic efficiency (*I*_*c*_/*I*_*d*_) changes versus H_2_O_2_ concentration; *I*_*c*_ and *I*_*d*_ are the cathodic peak currents in the presence and in the absence of hydrogen peroxide, respectively.

As can be observed, the catalytic efficiency increases with the H_2_O_2 _concentration up to 298 *μ*M, and then a plateau is reached. This is probably due to the denaturing effect of hydrogen peroxide at high concentration values.

Based on these results obtained using a classical GCE electrode and employing a very simple and easy immobilization procedure, the same modification system has been developed on screen-printed electrodes in view of a possible application for determination of hydrogen peroxide in real samples.

### 3.6. Morphological Characterization of Screen-Printed Electrodes and Electroanalytical and Kinetic Characterization

**Figure 5:**
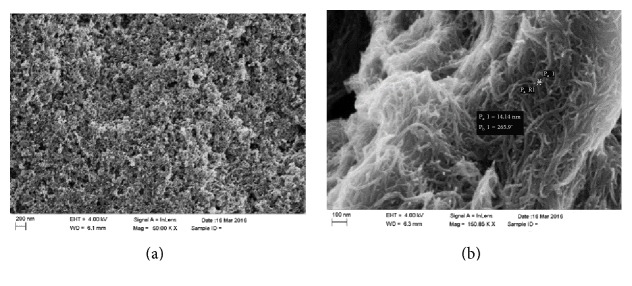
SEM images of electrodes surfaces: MG-SPE bare (a) and NAF-MWCNTs-COOH-MG-SPE modified electrode (b).

The surface morphology of the modified screen-printed electrodes (SPEs) was obtained by scanning electronic microscopy (SEM). In Figure 5(a), mesoporous graphite SPE (MG-SPE bare) surface, without modification, is shown.  Figure 5(b) reveals the presence of a cross-linked structure of multiwalled carbon nanotubes modified with carboxylic groups dispersed in a Nafion film (NAF-MWCNTs-COOH-MG-SPE surface). Moreover, the diameter of the carbon nanotubes (~14 nm) is indicated. In the presence of the enzyme, the highly porous architecture that is formed between the MWCNTs-COOH and the Nafion film is suitable for immobilization of catalase that is confirmed in the following electrochemical measures.

Also, electrochemical characterization of these SPEs was carried out and the results are reported in Table 4. Also, for these electrodes, the feature of nanomaterials to increase the sensor performances considerably is confirmed, so the following studies were performed using the NAF-MWCNTs-COOH-MG-SPE sensor.

Successively, the main electrochemical parameters of our proposed biosensor NAF-MWCNTs-COOH-MG-SPE-CAT were evaluated (see Table 5).

The electrochemical response of the obtained biosensor for different concentrations of H_2_O_2 _was studied. The current-concentration dependence of hydrogen peroxide was modeled by using Michaelis-Menten nonlinear fitting thus allowing the calculation of the main kinetic parameters; data obtained are reported in Table 6. It is clear that the biosensor has a good LOD of 0.83 *μ*mol L^−1^ and a good sensitivity to determine H_2_O_2 _concentrations. Moreover, a comparison of analytical and kinetic parameters for H_2_O_2 _detection for different redox protein modified electrodes is summarized in Table 6 [34, 81, 83, 85–94, 110, 114].

Also, the reproducibility of the developed biosensor was calculated as RSD = 5.0% by using 500 *μ*mol L^−1^ H_2_O_2_ in a series of six experiments. By the data achieved, the following can be assessed: (i) the immobilized enzyme retained good biocatalytic activity; (ii) the carbon nanotubes dispersed in the Nafion film provided an optimal microenvironment; (iii) the nanocomposite was a good matrix for catalase immobilization and biosensing preparation; (iv) the redox protein maintained active site accessibility and exchanged electrons with the sensor surface. This platform was applied for H_2_O_2_ sensing in real samples.

### 3.7. Determination of H_2_O_2_ in Beverages

Based on the results declared in the previous sections and in order to test the reliability of the proposed biosensor for practical application, different commercial beverages were chosen (tea, juice, and milk). Every sample was pretreated as reported in Section 2.3. The concentration of 15 *μ*mol L^−1^ was chosen because an FDA regulation currently limits residual H_2_O_2_ to 0.05 ppm (corresponding to 15 *μ*mol L^−1^), leached into distilled water, in finished food packages [115]. The results show good recoveries, in the range 100.3–105.7%, for our modified NAF-MWCNTs-COOH-MG-SPE-CAT biosensor (Table 7).

### 3.8. Stability of NAF-MWCNTs-COOH-MG-SPE-CAT Biosensor

The shelf lifetime of our modified biosensor was tested by measuring its current response obtained for 500 *μ*mol L^−1^ H_2_O_2_ concentration during a period of 21 days. The biosensor was stored in PBS buffer solution at 4°C before and after use. During the first week, a 4% decrease was observed, reaching a 15% decrease after three weeks. This result can be ascribable to the presence of the nanomaterials, which avoid the fouling phenomena of the surface which could affect the biosensor performances, and also the use of NAF/MWCNTs composite film provides a strong and biocompatible microenvironment for stabilizing the catalase activity.

## 4. Conclusion

In this study, an electrochemical biosensor was developed for the determination of hydrogen peroxide concentration in packaged beverages. To this aim, direct electrochemical properties of catalase, confined in a Nafion film on the surface of a glassy carbon electrode, were studied. The electron transfer coefficient, *α*, the electron transfer rate constant, *k*_*s*_, and the surface concentration of electroactive redox protein, Γ, were evaluated by cyclic voltammetry studies. The modification of the electrode surface by using nanostructured materials dispersed in Nafion polymeric solution resulted in an enhancement of the overall bioelectrochemical properties of the developed biosensor. The biocatalytic activity towards catalase substrate hydrogen peroxide confirmed that the immobilization procedure allowed a good microenvironment for catalase and facilitated the electron exchange to the electrode surface. Hence, based on these interesting results obtained, the same modification procedure was applied to screen-printed electrodes. Also, this platform of the modified biosensor was entirely characterized and was applied to detect H_2_O_2_ in spiked real samples of different commercial beverages obtaining good recoveries.

## Figures and Tables

**Table 1:** Electroactive area and heterogeneous standard rate constant of bare sensor and after modification steps.

Sensor	*A*_*e*_/mm^2^	*k* ^0^ × 10^−4^/cm s^−1^
Bare-GCE	4.89	9.3
NAF-GCE	2.16	6.5
NAF-MWCNTs-COOH-GCE	16.42	13.5

**Table 2:** Electrochemical parameters for immobilized catalase either in the absence or in the presence of nanomaterials.

Biosensor	*E* ^0′^/mV	*α*	*k*_*s*_/s^−1^	Γ/mol cm^−2^
NAF-GCE-CAT	−128	0.89	1.03	2.30 × 10^−10^
NAF-MWCNTs-GCE-CAT	−138	0.38	1.65	3.50 × 10^−10^
NAF-MWCNTs-COOH-GCE-CAT	−140	0.42	1.71	4.76 × 10^−10^

**Table 3:** Comparison of electrochemical parameters of the catalase modified glassy carbon electrodes by using MWCNTs recently developed for H_2_O_2_ determination.

Catalase modified GCE	*E* ^0′^/mV	*k*_*s*_/s^−1^	Γ/mol cm^−2^	Ref.
[bmim][PF_6_]-MWCNTs	~−100^a,d^	1.95	3.31 × 10^−10^	[75]
Ionic-liquid-MWCNTs-NH_2_	−460^a,d^	2.23	2.88 × 10^−10^	[76]
MWCNTs-NF-DTAB	−279^a,d^	10.71	2.6 × 10^−11^	[77]
CA-MWCNTs	−559^a,d^	1.22	1.49 × 10^−10^	[78]
PEI-MWCNTs-NF	−450^a,e^	1.05	2.10 × 10^−10^	[79]
MWCNTs-NF-DDAB	−380^a,c^	11.0	73 × 10^−12^	[80]
PLL-f-MWCNTs	−471^a,c^	5.48	4.072 × 10^−10^	[81]
NAF-MWCNTs-COOH-CYS-AuNPs	−441^a,d^	8.72	2 × 10^−9^	[82]
NAF-MWCNTs-COOH-GCE	−140^b,d^	1.71	4.76 × 10^−10^	This work

^a^Versus Ag/AgCl; ^b^versus SCE; ^c^pH 6.5; ^d^pH 7.0; ^e^pH 7.5.

**Table 4:** Electroactive area and heterogeneous standard rate constant of bare screen-printed sensor and after the modification step.

Sensor-SPE	*A*_*e*_/mm^2^	*k* ^0^ × 10^−4^/cm s^−1^
MG-SPE bare	7.93	16.5
NAF-MWCNTs-COOH-MG-SPE	11.65	30.2

**Table 5:** Electrochemical parameters for immobilized catalase in the presence of nanomaterials on mesoporous graphite SPE.

Biosensor	*E* ^0′^/mV	*α*	*k*_*s*_/s^−1^	Γ/mol cm^−2^
NAF-MWCNTs-COOH-MG-SPE-CAT	−254	0.37	0.60	2.87 × 10^−10^

**Table 6:** Comparison of analytical and kinetic parameters for H_2_O_2_ detection for different redox protein modified electrodes using H_2_O_2_ as substrate.

*K*_*M*_^app^, mmol L^−1^	Slope, *μ*A *μ*mol^−1^ L	Linear range, *μ*mol L^−1^	LOD, *μ*mol L^−1^	*R*	Ref.
0.26	0.0112	0.21–3000	0.08	0.999	[34]
0.21	287.98	10–3200	3.33	0.995	[78]
0.224	0.392	1–3600	0.008	0.998	[81]
—	—	200–5000	1.0	0.997	[83]
2.61	—	5–5130	1.7	0.999	[84]
—	—	10–1130	0.65		[85]
0.51	369.2	6–1010	0.39	0.996	[86]
51.7	—	0.0067–8000	0.0022	0.998	[87]
—	—	9.8–6000	4.9	0.999	[88]
—	0.9103	0.1–100	0.05	0.997	[89]
—	0.61	0.3–1000	0.1	0.999	[88]
0.21	0.0281	1–140	0.93	0.998	[90]
0.29	0.315	50–1800	4.0	0.997	[91]
0.010		1–600	7.3		[92]
0.089		50–135	1.67		[93]
2.81		0.3–600	0.05		[94]
1.5	0.38	2.5–1150	0.83	0.999	This work

**Table 7:** Determination of H_2_O_2_ in several commercial beverages, spiked with H_2_O_2_ 15 *μ*mol L^−1^, using NAF-MWCNTs-COOH-MG-SPE-CAT as biosensor.

Beverages samples	Found/*μ*mol L^−1^	Recovery %
Peach tea	15.9	105.7
Lemon tea	15.3	102.3
Green tea	14.8	101.0
Apple juice	14.9	100.3
Blood orange juice	15.7	104.8
Pineapple juice	14.7	102.0
Lactose-free milk	15.6	103.8

## References

[1] Wang J., Lin Y., Chen L. (1993). Organic-phase biosensors for monitoring phenol and hydrogen peroxide in pharmaceutical antibacterial products. *The Analyst*.

[2] Pournaghi-Azar M. H., Ahour F., Pournaghi-Azar F. (2010). Simple and rapid amperometric monitoring of hydrogen peroxide in salivary samples of dentistry patients exploiting its electro-reduction on the modified/palladized aluminum electrode as an improved electrocatalyst. *Sensors and Actuators B: Chemical*.

[3] Lin Y., Cui X., Li L. (2005). Low-potential amperometric determination of hydrogen peroxide with a carbon paste electrode modified with nanostructured cryptomelane-type manganese oxides. *Electrochemistry Communications*.

[4] Ping J., Wu J., Fan K., Ying Y. (2011). An amperometric sensor based on Prussian blue and poly(o-phenylenediamine) modified glassy carbon electrode for the determination of hydrogen peroxide in beverages. *Food Chemistry*.

[5] Alpat Ş., Alpat S. K., Dursun Z., Telefoncu A. (2009). Development of a new biosensor for mediatorless voltammetric determination of hydrogen peroxide and its application in milk samples. *Journal of Applied Electrochemistry*.

[6] Hsu C.-L., Chang K.-S., Kuo J.-C. (2008). Determination of hydrogen peroxide residues in aseptically packaged beverages using an amperometric sensor based on a palladium electrode. *Food Control*.

[7] International Agency for the Reaserch on Cancer (IARC) (1999). *Hydrogen Peroxide*.

[8] World Health Organization (WHO) (1973). *Hydrogen Peroxide, 267. Joint FAO/WHO Expert Committee on Food Additives*.

[9] Canadian Centre for Occupational Health and Safety (CCOHS) (1998). *Cheminfo: Hydrogen Peroxide Solutions 35% and Greater. Record Number 198*.

[10] International Programme on Chemical Safety (IPCS) (2000). Hydrogen peroxide (>60% solution in water). *International Chemical Safety Card*.

[11] Kok G. L., Holler T. P., Lopez M. B., Nachtrieb H. A., Yuan M. (1978). Chemiluminescent method for determination of hydrogen peroxide in the ambient atmosphere. *Environmental Science and Technology*.

[12] He S., Shi W., Zhang X., Li J., Huang Y. (2010). *β*-Cyclodextrins-based inclusion complexes of CoFe_2_O_4_ magnetic nanoparticles as catalyst for the luminol chemiluminescence system and their applications in hydrogen peroxide detection. *Talanta*.

[13] Yamashiro N., Uchida S., Satoh Y. (2004). Determination of hydrogen peroxide in water by chemiluminescence detection, (I) flow injection type hydrogen peroxide detection system. *Journal of Nuclear Science and Technology*.

[14] Rocha F. R. P., Ródenas-Torralba E., Reis B. F., Morales-Rubio Á., De La Guardia M. (2005). A portable and low cost equipment for flow injection chemiluminescence measurements. *Talanta*.

[15] Zhou G.-J., Wang G., Xu J.-J., Chen H.-Y. (2002). Reagentless chemiluminescence biosensor for determination of hydrogen peroxide based on the immobilization of horseradish peroxidase on biocompatible chitosan membrane. *Sensors and Actuators, B: Chemical*.

[16] Hu X., Han H., Hua L., Sheng Z. (2010). Electrogenerated chemiluminescence of blue emitting ZnSe quantum dots and its biosensing for hydrogen peroxide. *Biosensors and Bioelectronics*.

[17] Lu S., Song J., Campbell-Palmer L. (2009). A modified chemiluminescence method for hydrogen peroxide determination in apple fruit tissues. *Scientia Horticulturae*.

[18] Genfa Z., Dasgupta P. K., Edgemond W. S., Marx J. N. (1991). Determination of hydrogen peroxide by photoinduced fluorogenic reactions. *Analytica Chimica Acta*.

[19] Lazrus A. L., Kok G. L., Gitlin S. N., Lind J. A., McLaren S. E. (1985). Automated fluorometric method for hydrogen peroxide in atmospheric precipitation. *Analytical Chemistry*.

[20] Albers A. E., Okreglak V. S., Chang C. J. (2006). A FRET-based approach to ratiometric fluorescence detection of hydrogen peroxide. *Journal of the American Chemical Society*.

[21] He F., Tang Y., Yu M., Wang S., Li Y., Zhu D. (2006). Fluorescence-amplifying detection of hydrogen peroxide with cationic conjugated polymers, and its application to glucose sensing. *Advanced Functional Materials*.

[22] Hurdis E. C., Romeyn H. (1954). Accuracy of determination of hydrogen peroxide by cerate oxidimetry. *Analytical Chemistry*.

[23] Prasada Rao M. S., Mohan Rao A. R., Ramana K. V., Sagi S. R. (1990). Thallimetric oxidations-V: titrimetric and spectrophotometric determination of hydrogen peroxide. *Talanta*.

[24] Lobnik A., Ajlakovi M. (2001). Sol-gel based optical sensor for continuous determination of dissolved hydrogen peroxide. *Sensors and Actuators, B: Chemical*.

[25] Sunil K., Narayana B. (2008). Spectrophotometric determination of hydrogen peroxide in water and cream samples. *Bulletin of Environmental Contamination and Toxicology*.

[26] Zhang K., Mao L., Cai R. (2000). Stopped-flow spectrophotometric determination of hydrogen peroxide with hemoglobin as catalyst. *Talanta*.

[27] Tarvin M., McCord B., Mount K., Sherlach K., Miller M. L. (2010). Optimization of two methods for the analysis of hydrogen peroxide: high performance liquid chromatography with fluorescence detection and high performance liquid chromatography with electrochemical detection in direct current mode. *Journal of Chromatography A*.

[28] Bai Y.-H., Du Y., Xu J.-J., Chen H.-Y. (2007). Choline biosensors based on a bi-electrocatalytic property of MnO_2_ nanoparticles modified electrodes to H_2_O_2_. *Electrochemistry Communications*.

[29] Hamidi H., Shams E., Yadollahi B., Esfahani F. K. (2009). Fabrication of carbon paste electrode containing [PFeW_11_O_39_]^4−^ polyoxoanion supported on modified amorphous silica gel and its electrocatalytic activity for H_2_O_2_ reduction. *Electrochimica Acta*.

[30] Lo P.-H., Kumar S. A., Chen S.-M. (2008). Amperometric determination of H_2_O_2_ at nano-TiO_2_/DNA/thionin nanocomposite modified electrode. *Colloids and Surfaces B: Biointerfaces*.

[31] Tseng K.-S., Chen L.-C., Ho K.-C. (2005). Amperometric detection of hydrogen peroxide at a Prussian Blue-modified FTO electrode. *Sensors and Actuators B: Chemical*.

[32] Xu Y., Peng W., Liu X., Li G. (2004). A new film for the fabrication of an unmediated H_2_O_2_ biosensor. *Biosensors and Bioelectronics*.

[33] Guascito M. R., Filippo E., Malitesta C., Manno D., Serra A., Turco A. (2008). A new amperometric nanostructured sensor for the analytical determination of hydrogen peroxide. *Biosensors and Bioelectronics*.

[34] Chen S., Yuan R., Chai Y., Zhang L., Wang N., Li X. (2007). Amperometric third-generation hydrogen peroxide biosensor based on the immobilization of hemoglobin on multiwall carbon nanotubes and gold colloidal nanoparticles. *Biosensors and Bioelectronics*.

[35] Shamsipur M., Kazemi S. H., Mousavi M. F. (2008). Impedance studies of a nano-structured conducting polymer and its application to the design of reliable scaffolds for impedimetric biosensors. *Biosensors and Bioelectronics*.

[36] Santhosh P., Manesh K. M., Gopalan A., Lee K.-P. (2006). Fabrication of a new polyaniline grafted multi-wall carbon nanotube modified electrode and its application for electrochemical detection of hydrogen peroxide. *Analytica Chimica Acta*.

[37] Yang G., Chen F., Yang Z. (2012). Electrocatalytic oxidation of hydrogen peroxide based on the shuttlelike nano-CuO-modified electrode. *International Journal of Electrochemistry*.

[38] Zhu S., Fan L., Liu X. (2008). Determination of concentrated hydrogen peroxide at single-walled carbon nanohorn paste electrode. *Electrochemistry Communications*.

[39] Guascito M. R., Chirizzi D., Malitesta C. (2011). Low-potential sensitive H_2_O_2_ detection based on composite micro tubular Te adsorbed on platinum electrode. *Biosensors and Bioelectronics*.

[40] Sanford A. L., Morton S. W., Whitehouse K. L. (2010). Voltammetric detection of hydrogen peroxide at carbon fiber microelectrodes. *Analytical Chemistry*.

[41] Liu M., Liu R., Chen W. (2013). Graphene wrapped Cu_2_O nanocubes: non-enzymatic electrochemical sensors for the detection of glucose and hydrogen peroxide with enhanced stability. *Biosensors and Bioelectronics*.

[42] Song M.-J., Hwang S. W., Whang D. (2010). Non-enzymatic electrochemical CuO nanoflowers sensor for hydrogen peroxide detection. *Talanta*.

[43] Ju J., Chen W. (2015). In situ growth of surfactant-free gold nanoparticles on nitrogen-doped graphene quantum dots for electrochemical detection of hydrogen peroxide in biological environments. *Analytical Chemistry*.

[44] Thenmozhi K., Narayanan S. S. (2007). Electrochemical sensor for H_2_O_2_ based on thionin immobilized 3-aminopropyltrimethoxy silane derived sol-gel thin film electrode. *Sensors and Actuators, B: Chemical*.

[45] Upadhyay A. K., Ting T.-W., Chen S.-M. (2009). Amperometric biosensor for hydrogen peroxide based on coimmobilized horseradish peroxidase and methylene green in ormosils matrix with multiwalled carbon nanotubes. *Talanta*.

[46] Zhao W., Xu J.-J., Chen H.-Y. (2006). Electrochemical biosensors based on layer-by-layer assemblies. *Electroanalysis*.

[47] Chandra S., Lokesh K. S., Nicolai A., Lang H. (2009). Dendrimer-rhodium nanoparticle modified glassy carbon electrode for amperometric detection of hydrogen peroxide. *Analytica Chimica Acta*.

[48] Lu Q., Dong X., Li L.-J., Hu X. (2010). Direct electrochemistry-based hydrogen peroxide biosensor formed from single-layer graphene nanoplatelet-enzyme composite film. *Talanta*.

[49] Song Y., Wang L., Ren C., Zhu G., Li Z. (2006). A novel hydrogen peroxide sensor based on horseradish peroxidase immobilized in DNA films on a gold electrode. *Sensors and Actuators, B: Chemical*.

[50] Ting S. W., Periasamy A. P., Chen S.-M., Saraswathi R. (2011). Direct electrochemistry of catalase immobilized at electrochemically reduced graphene oxide modified electrode for amperometric H_2_O_2_ biosensor. *International Journal of Electrochemical Science*.

[51] Karyakin A. A., Karyakina E. E., Gorton L. (2000). Amperometric biosensor for glutamate using prussian blue-based ‘artificial peroxidase’ as a transducer for hydrogen peroxide. *Analytical Chemistry*.

[52] Gao F., Yuan R., Chai Y., Chen S., Cao S., Tang M. (2007). Amperometric hydrogen peroxide biosensor based on the immobilization of HRP on nano-Au/Thi/poly (p-aminobenzene sulfonic acid)-modified glassy carbon electrode. *Journal of Biochemical and Biophysical Methods*.

[53] Majidi M. R., Pournaghi-Azar M. H., Saadatirad A., Alipour E. (2015). Simple and rapid amperometric monitoring of hydrogen peroxide at hemoglobin-modified pencil lead electrode as a novel biosensor: application to the analysis of honey sample. *Food Analytical Methods*.

[54] Zong S., Cao Y., Zhou Y., Ju H. (2007). Hydrogen peroxide biosensor based on hemoglobin modified zirconia nanoparticles-grafted collagen matrix. *Analytica Chimica Acta*.

[55] Nasirizadeh N., Hajihosseini S., Shekari Z., Ghaani M. (2015). A novel electrochemical biosensor based on a modified gold electrode for hydrogen peroxide determination in different beverage samples. *Food Analytical Methods*.

[56] Melik-Adamyan W. R., Barynin V. V., Vagin A. A. (1986). Comparison of beef liver and *Penicillium vitale* catalases. *Journal of Molecular Biology*.

[57] Murthy M. R. N., Reid T. J., Sicignano A., Tanaka N., Rossmann M. G. (1981). Structure of beef liver catalase. *Journal of Molecular Biology*.

[58] Borges P. T., Frazão C., Miranda C. S., Carrondo M. A., Romão C. V. (2014). Structure of the monofunctional heme catalase DR1998 from *Deinococcus radiodurans*. *The FEBS journal*.

[59] Díaz A., Loewen P. C., Fita I., Carpena X. (2012). Thirty years of heme catalases structural biology. *Archives of Biochemistry and Biophysics*.

[60] Shamsipur M., Asgari M., Maragheh M. G., Moosavi-Movahedi A. A. (2012). A novel impedimetric nanobiosensor for low level determination of hydrogen peroxide based on biocatalysis of catalase. *Bioelectrochemistry*.

[61] Pakhomova S., Gao B., Boeglin W. E., Brash A. R., Newcomer M. E. (2009). The structure and peroxidase activity of a 33-kDa catalase-related protein from *Mycobacterium avium ssp. Paratuberculosis*. *Protein Science*.

[62] Melik-Adamyan W., Bravo J., Carpena X. (2001). Substrate flow in catalases deduced from the crystal structures of active site variants of HPII from *Escherichia coli*. *Proteins: Structure, Function and Genetics*.

[63] Salimi A., Noorbakhsh A., Ghadermarz M. (2005). Direct electrochemistry and electrocatalytic activity of catalase incorporated onto multiwall carbon nanotubes-modified glassy carbon electrode. *Analytical Biochemistry*.

[64] Zhou H., Lu T.-H., Shi H.-X., Dai Z.-H., Huang X.-H. (2008). Direct electrochemistry and electrocatalysis of catalase immobilized on multi-wall carbon nanotubes modified glassy carbon electrode and its application. *Journal of Electroanalytical Chemistry*.

[65] Salimi A., Noorbakhsh A., Ghadermarzi M. (2007). Amperometric detection of nitrite, iodate and periodate at glassy carbon electrode modified with catalase and multi-wall carbon nanotubes. *Sensors and Actuators, B: Chemical*.

[66] Zhao G.-C., Yin Z.-Z., Zhang L., Wei X.-W. (2005). Direct electrochemistry of cytochrome c on a multi-walled carbon nanotubes modified electrode and its electrocatalytic activity for the reduction of H_2_O_2_. *Electrochemistry Communications*.

[67] Tortolini C., Rea S., Carota E., Cannistraro S., Mazzei F. (2012). Influence of the immobilization procedures on the electroanalytical performances of Trametes versicolor laccase based bioelectrode. *Microchemical Journal*.

[68] Journet C., Maser W. K., Bernier P. (1997). Large-scale production of single-walled carbon nanotubes by the electric-arc technique. *Nature*.

[69] Star A., Stoddart J. F., Steuerman D. (2001). Preparation and properties of polymer-wrapped single-walled carbon nanotubes. *Angewandte Chemie—International Edition*.

[70] Zhang W., Suhr J., Koratkar N. (2006). Carbon nanotube/polycarbonate composites as multifunctional strain sensors. *Journal of Nanoscience and Nanotechnology*.

[71] Liu C., Choi J. (2012). Improved Dispersion of Carbon Nanotubes in Polymers at High Concentrations. *Nanomaterials*.

[72] Wang J., Musameh M., Lin Y. (2003). Solubilization of carbon nanotubes by Nafion toward the preparation of amperometric biosensors. *Journal of the American Chemical Society*.

[73] Andrieux C. P., Audebert P., Divisia-Blohorn B., Aldebert P., Michalak F. (1990). Electrochemistry in hydrophobic Nafion gels: part 1. Electrochemical behaviour of electrodes modified by hydrophobic Nafion gels loaded with ferrocenes. *Journal of Electroanalytical Chemistry*.

[74] Liu H., Deng J. (1995). An amperometric lactate sensor employing tetrathiafulvalene in Nafion film as electron shuttle. *Electrochimica Acta*.

[75] Prakash P. A., Yogeswaran U., Chen S.-M. (2009). A review on direct electrochemistry of catalase for electrochemical sensors. *Sensors*.

[76] Rahimi P., Rafiee-Pour H.-A., Ghourchian H., Norouzi P., Ganjali M. R. (2010). Ionic-liquid/NH2-MWCNTs as a highly sensitive nano-composite for catalase direct electrochemistry. *Biosensors and Bioelectronics*.

[77] Hashemnia S., Khayatzadeh S., Moosavi-Movahedi A. A., Ghourchian H. (2011). Direct electrochemistry of catalase in multiwall carbon nanotube/dodecyl trimethylammonium bromide film covered with a layer of nafion on a glassy carbon electrode. *International Journal of Electrochemical Science*.

[78] Periasamy A. P., Ho Y.-H., Chen S.-M. (2011). Multiwalled carbon nanotubes dispersed in carminic acid for the development of catalase based biosensor for selective amperometric determination of H_2_O_2_ and iodate. *Biosensors and Bioelectronics*.

[79] Vatsyayan P., Bordoloi S., Goswami P. (2010). Large catalase based bioelectrode for biosensor application. *Biophysical Chemistry*.

[80] Arun Prakash P., Yogeswaran U., Chen S.-M. (2009). Direct electrochemistry of catalase at multiwalled carbon nanotubes-nafion in presence of needle shaped DDAB for H_2_O_2_ sensor. *Talanta*.

[81] Ezhil Vilian A. T., Chen S.-M., Lou B.-S. (2014). A simple strategy for the immobilization of catalase on multi-walled carbon nanotube/poly (L-lysine) biocomposite for the detection of H_2_O_2_ and iodate. *Biosensors and Bioelectronics*.

[82] Hong J., Yang W.-Y., Zhao Y.-X. (2013). Catalase immobilized on a functionalized multi-walled carbon nanotubes-gold nanocomposite as a highly sensitive bio-sensing system for detection of hydrogen peroxide. *Electrochimica Acta*.

[83] Wang Y., Li T., Zhang W., Huang Y. (2014). A hydrogen peroxide biosensor with high stability based on gelatin-multiwalled carbon nanotubes modified glassy carbon electrode. *Journal of Solid State Electrochemistry*.

[84] Zhou K., Zhu Y., Yang X., Luo J., Li C., Luan S. (2010). A novel hydrogen peroxide biosensor based on Au-graphene-HRP-chitosan biocomposites. *Electrochimica Acta*.

[85] Tangkuaram T., Ponchio C., Kangkasomboon T., Katikawong P., Veerasai W. (2007). Design and development of a highly stable hydrogen peroxide biosensor on screen printed carbon electrode based on horseradish peroxidase bound with gold nanoparticles in the matrix of chitosan. *Biosensors and Bioelectronics*.

[86] Feng Q., Liu K., Fu J. (2012). irect electrochemistry of hemoglobin based on nano-composite film of gold nanopaticles and poly (diallyldimethylammonium chloride) functionalized graphene. *Electrochimica Acta*.

[87] Mao C.-J., Chen X.-B., Niu H.-L., Song J.-M., Zhang S.-Y., Cui R.-J. (2012). A novel enzymatic hydrogen peroxide biosensor based on Ag/C nanocables. *Biosensors and Bioelectronics*.

[88] Li W.-T., Wang M.-H., Li Y.-J., Sun Y., Li J.-C. (2011). Linker-free layer-by-layer self-assembly of gold nanoparticle multilayer films for direct electron transfer of horseradish peroxidase and H_2_O_2_ detection. *Electrochimica Acta*.

[89] Kang X. B., Pang G. C., Liang X. Y., Wang M., Liu J., Zhu W. M. (2012). Study on a hydrogen peroxide biosensor based on horseradish peroxidase/GNPs-thionine/chitosan. *Electrochimica Acta*.

[90] Xuan J., Jia X.-D., Jiang L.-P., Abdel-Halim E. S., Zhu J.-J. (2012). Gold nanoparticle-assembled capsules and their application as hydrogen peroxide biosensor based on hemoglobin. *Bioelectrochemistry*.

[91] Tan X.-C., Zhang J.-L., Tan S.-W. (2009). Amperometric hydrogen peroxide biosensor based on immobilization of hemoglobin on a glassy carbon electrode modified with Fe_3_O_4_/chitosan core-shell microspheres. *Sensors*.

[92] Gao Y.-C., Xi K., Wang W.-N., Jia X.-D., Zhu J.-J. (2011). A novel biosensor based on a gold nanoflowers/hemoglobin/carbon nanotubes modified electrode. *Analytical Methods*.

[93] Zhu W.-L., Wang Y., Xuan J., Zhang J.-R. (2011). Fabrication of a novel hydrogen peroxide biosensor based on C@Au composite. *Journal of Nanoscience and Nanotechnology*.

[94] Huang K.-J., Niu D.-J., Liu X. (2011). Direct electrochemistry of catalase at amine-functionalized graphene/gold nanoparticles composite film for hydrogen peroxide sensor. *Electrochimica Acta*.

[95] Bard A. J., Faulkner L. R. (2001). *Electrochemical Methods: Fundamentals and Applications*.

[96] Lavagnini I., Antiochia R., Magno F. (2004). An extended method for the practical evaluation of the standard rate constant from cyclic voltammetric data. *Electroanalysis*.

[97] Nicholson R. S. (1965). Theory and application of cyclic voltammetry for measurement of electrode reaction kinetics. *Analytical Chemistry*.

[98] Klingler R. J., Kochi J. K. (1981). Electron-transfer kinetics from cyclic voltammetry. Quantitative description of electrochemical reversibility. *Journal of Physical Chemistry*.

[99] Wang J. (2005). Carbon-nanotube based electrochemical biosensors: a review. *Electroanalysis*.

[100] Coleman J. N., Khan U., Blau W. J., Gun'ko Y. K. (2006). Small but strong: a review of the mechanical properties of carbon nanotube-polymer composites. *Carbon*.

[101] Wang J. (2005). Nanomaterial-based electrochemical biosensors. *Analyst*.

[102] Yáñez-Sedeño P., Pingarrón J. M., Riu J., Rius F. X. (2010). Electrochemical sensing based on carbon nanotubes. *TrAC—Trends in Analytical Chemistry*.

[103] Yang W., Ratinac K. R., Ringer S. R., Thordarson P., Gooding J. J., Braet F. (2010). Carbon nanomaterials in biosensors: should you use nanotubes or graphene?. *Angewandte Chemie—International Edition*.

[104] De Volder M. F. L., Tawfick S. H., Baughman R. H., Hart A. J. (2013). Carbon nanotubes: present and future commercial applications. *Science*.

[105] Sanzó G., Tortolini C., Antiochia R., Favero G., Mazzei F. (2015). Development of carbon-based nano-composite materials for direct electron transfer based biosensors. *Journal of Nanoscience and Nanotechnology*.

[106] Zhang Z., Chouchane S., Magliozzo R. S., Rusling J. F. (2002). Direct voltammetry and catalysis with Mycobacterium tuberculosis catalase-peroxidase, peroxidases, and catalase in lipid films. *Analytical Chemistry*.

[107] Yamazaki I., Araiso T., Hayashi Y., Yamada H., Makino R. (1978). Analysis of acid-base properties of peroxidase and myoglobin. *Advances in Biophysics*.

[108] Hashemnia S., Ghourchian H., Moosavi-Movahedi A. A., Faridnouri H. (2009). Direct electrochemistry of chemically modified catalase immobilized on an oxidatively activated glassy carbon electrode. *Journal of Applied Electrochemistry*.

[109] Laviron E. (1979). General expression of the linear potential sweep voltammogram in the case of diffusionless electrochemical systems. *Journal of Electroanalytical Chemistry*.

[110] Lu H., Li Z., Hu N. (2003). Direct voltammetry and electrocatalytic properties of catalase incorporated in polyacrylamide hydrogel films. *Biophysical Chemistry*.

[111] Chen X., Ferrigno R., Yang J., Whitesides G. M. (2002). Redox properties of cytochrome *c* adsorbed on self-assembled monolayers: a probe for protein conformation and orientation. *Langmuir*.

[112] Vostiar I., Ferapontova E. E., Gorton L. (2004). Electrical 'wiring' of viable *Gluconobacter oxydans* cells with a flexible osmium-redox polyelectrolyte. *Electrochemistry Communications*.

[113] Gorton L., Lindgren A., Larsson T., Munteanu F. D., Ruzgas T., Gazaryan I. (1999). Direct electron transfer between heme-containing enzymes and electrodes as basis for third generation biosensors. *Analytica Chimica Acta*.

[114] Wang W., Zhang T.-J., Zhang D.-W. (2011). Amperometric hydrogen peroxide biosensor based on the immobilization of heme proteins on gold nanoparticles-bacteria cellulose nanofibers nanocomposite. *Talanta*.

[115] Code of Federal Regulations (2000). *Indirect Food Additivies: Adjuvants, Production Aids and Sanitizers. 21 CFR 178.1005. Office of the Federal Register*.

